# Changes in the microglial phenotype drive neuroinflammation independent of systemic inflammation in the acute stage of heatstroke

**DOI:** 10.1016/j.isci.2026.115254

**Published:** 2026-03-06

**Authors:** Ping Li, Zeze Wang, Jun Liu, Gong Wang, Xue Luo, Zhen Luo, Tingting Shen, Genlin He, Xuesen Yang

**Affiliations:** 1 Department of Tropical Medicine, Army Medical University, Chongqing, China; 2 Department of Neurology, Xinqiao Hospital, Army Medical University, Chongqing, China

**Keywords:** health sciences

## Abstract

Heatstroke causes acute injury and damage across numerous organ systems, during this process, inflammation drives disease progression. In this study, we correlated temporal changes in inflammatory response with barrier breakdown within 24 h following heatstroke onset to explore whether inflammation in circulation can influence inflammation in brain. We found both pro-inflammatory cytokines expression and pathological lesions in tissue were elevated at 1 h and 6 h after heatstroke onset. However, by 24 h after heatstroke onset, while systemic inflammation was sustained at high levels, neuroinflammation and cerebral cortex damage had begun to reverse. The change in microglial phenotype from classic activation at 1 h to alternative activation at 24 h post heatstroke may explain the subsequent abatement of neuroinflammation. Taken together, our results may indicate microglial phenotype transformation drives neuroinflammation independent of systemic inflammation during the acute stage of heatstroke.

## Highlights

• Systemic inflammation affects neuroinflammation within 6 h after heatstroke onset
• Neuroinflammation develops independent of circulation inflammation at 24 h post heatstroke
• Microglial phenotype transformation drives neuroinflammation 24 h post-heatstroke

## Graphical abstract

**Figure:**
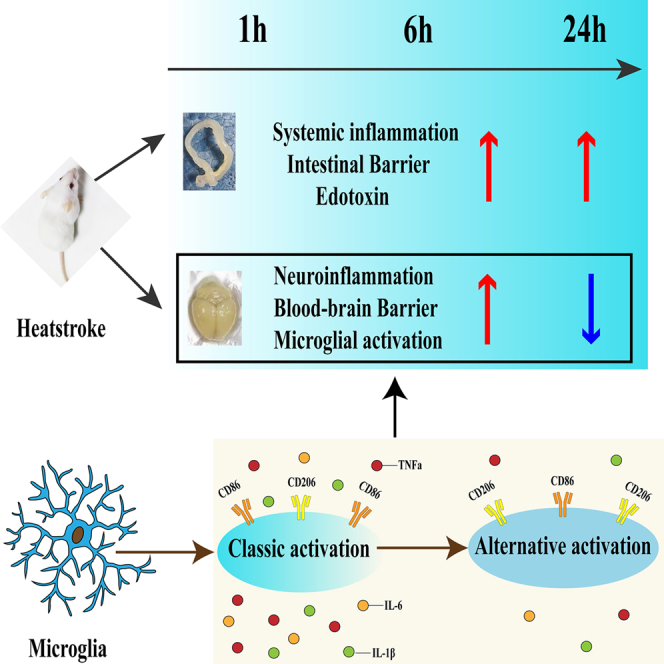

## Introduction

Heatstroke is a severe life-threatening illness characterized by a rapid increase in core body temperature above 40°C and central nervous system dysfunction, such as delirium, convulsions, and coma.^1^ Although physical cooling is an effective measure for reducing heatstroke-induced injury, severe central nervous system dysfunction, high levels of circulating inflammatory cytokines, and multiple organ failure caused by high temperature are still challenging to treat in hospitals.^2^^,^^3^^,^^4^ A total of 1714 heatstroke-induced deaths were reported in the United States in 2022, and 91467 ambulance transports for heatstroke patients were called in Japan in 2023 because of climate change.^5^^,^^6^ The lack of a specific or effective clinical treatment for heatstroke is the critical reason for its high fatality rate. With global warming, the frequency and intensity of heat waves are increasing, and heatstroke will become the main cause of death worldwide. Elucidating the pathogenesis mechanism at the integral level may help to identify new interventional targets for heatstroke.

Multiorgan system failure is the ultimate reason of death in heatstroke patients, whereas excessive inflammation is the main cause. As a critical pathophysiological mechanism, systemic inflammatory response syndrome (SIRS) is commonly regarded as a pivotal factor in the progression of heatstroke-induced multiple organ dysfunction syndrome (MODS). Increased circulating levels of TNF-α, IL-6, and IL-1β are correlated with lesions in the heart, liver, kidney, lung, spleen, and intestine, whereas alterations in the levels of these pro-inflammatory cytokines through anti-inflammatory drugs or receptor antagonists can alleviate multiple organ damage and improve survival in heatstroke mice.^7^^,^^8^^,^^9^^,^^10^ Dysfunction in the central nervous system occurs during the early stage of heatstroke, preceding damage to other organ systems. Neuroinflammation functions as a magnifier in heatstroke-induced brain injury, as a severe inflammatory response in the central nervous system is often correlated with serious neurological damage and a poor prognosis in heatstroke patients.^11^ Previous studies have shown that inflammation in circulation is correlated with inflammation in the central nervous system in many diseases.^12^ However, how peripheral and central nervous system inflammation develops and whether neuroinflammation can be influenced by peripheral inflammation during heatstroke recovery is still unknown.

As the critical hubs for maintaining the stability of the peripheral and central environment, the gut barrier and blood-brain barrier play important roles in restraining the penetration of inflammation-related harmful substances in the intestine and blood into the systemic circulation and brain, respectively, under physiological and pathological conditions. However, both barriers have been reported to be compromised upon high-temperature exposure. The permeability of the intestine can change after exposure to a moderate temperature (approximately 39°C) for just 1–2 h; thereafter, the endotoxin and bacteria will leak into the blood through the breached barrier, resulting in an inflammatory response and stimulation of inflammatory mediators in the systemic circulation.^5^^,^^13^ Alterations in the blood-brain barrier can also be observed at temperatures above 38°C^14^ which expose the brain to systemic and intestinal toxins, such as LPS,^5^ ultimately leading to the aggravation of neuroinflammation and brain injury during heatstroke. Disruption of the blood-brain barrier also mediates neuroinflammation by affecting the state of microglia, which are the resident immune cells in the brain. Microglial activation has been shown to trigger neuroinflammation and result in neurotoxicity and neuronal dysfunction during heatstroke.^11^^,^^15^^,^^16^ Although many studies related to the intestinal barrier and blood-brain barrier during heatstroke occurrence have been conducted, little is known about the status of these two barriers during the recovery period after heatstroke.

In this study, we established a heatstroke model in mice to investigate the development of inflammatory responses in both the central nervous system and the periphery within 24 h following heatstroke onset. In addition, as the main gates of the intestine and brain, the intestinal barrier and blood-brain barrier were investigated in this study because their integrity plays a critical role in mediating the inflammatory response in both the circulation and the brain.

## Results

### Establishment of a heatstroke model in mice

**Figure 1:**
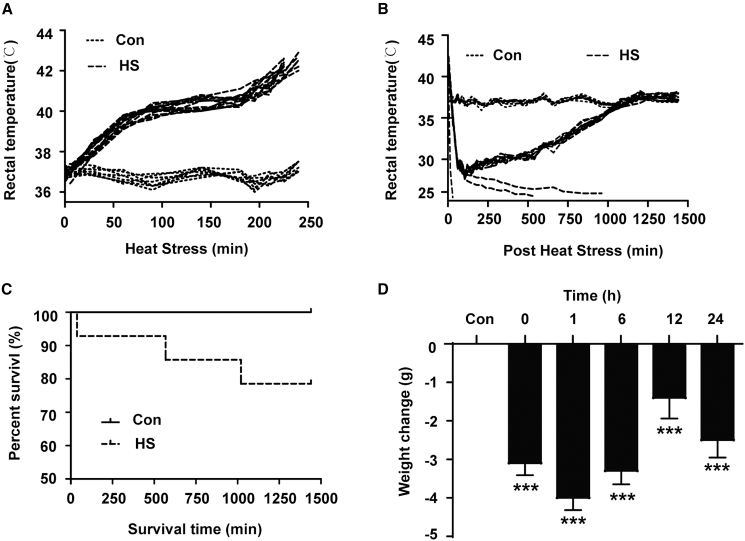
Establishment of a heatstroke model in mice The mice were exposed to 41.2 ± 0.5°C ambient temperature until their rectal temperature reached 42.4°C, and then they were allowed to recover at an ambient temperature of 25 ± 0.5°C for the indicated times. Rectal temperature during heat stress (A) and post heat stress (B), survival curves (C) and body weight changes (D) were recorded. The data are expressed as the mean ± SEM (*n* ≥ 6 per group). ∗*p* < 0.05, ∗∗*p* < 0.01, ∗∗∗*p* < 0.001, vs. Con.

First, we established a heatstroke model in mice. The mice were placed in a controlled climate chamber (41.2 ± 0.5°C, 60 ± 2%) to receive heat stress treatment, and their rectal temperature was recorded. Consistent with previous studies, the temperature profile of mice subjected to heat stress showed an initial overshoot, followed by a slow and stable change for approximately 60 min (starting from 120 ± 18 min to 180 ± 12 min during heat stress), after which it quickly increased to 42.4 ± 0.2°C at approximately 240 min (Figure 1A). The rectal temperature of the mice in the control group was steadily maintained at 37.5 ± 0.5°C during the experiment (Figure 1A). All the mice whose rectal temperature was above 42.4 were defined as experiencing the onset of heatstroke and were removed from the heat conditions to a 25 ± 0.5°C environment to recover, and their rectal temperature was recorded during the recovery stage. The results revealed that the rectal temperature reached the lowest value (28.0 ± 1.5°C) within 2 h after the mice were removed from the heat conditions; then, the rectal temperature gradually increased and returned to normal which could compare to the temperature in the normothermic group until 24 h (Figure 1B). Mortality data were collected during the whole heat stress and recovery period, as it demonstrated no mice died in the control group, while the mortality rate was 21.43% in the heatstroke group (Figure 1C). The body weight change was calculated and was significantly lower in the groups that recovered from heat stress for 1 h, 6 h, 12 h, and 24 h than in the control group (Figure 1D). All these results suggest we successfully established a heatstroke model in mice.

### Time course of multiorgan injury during the acute stage of heatstroke

**Figure 2:**
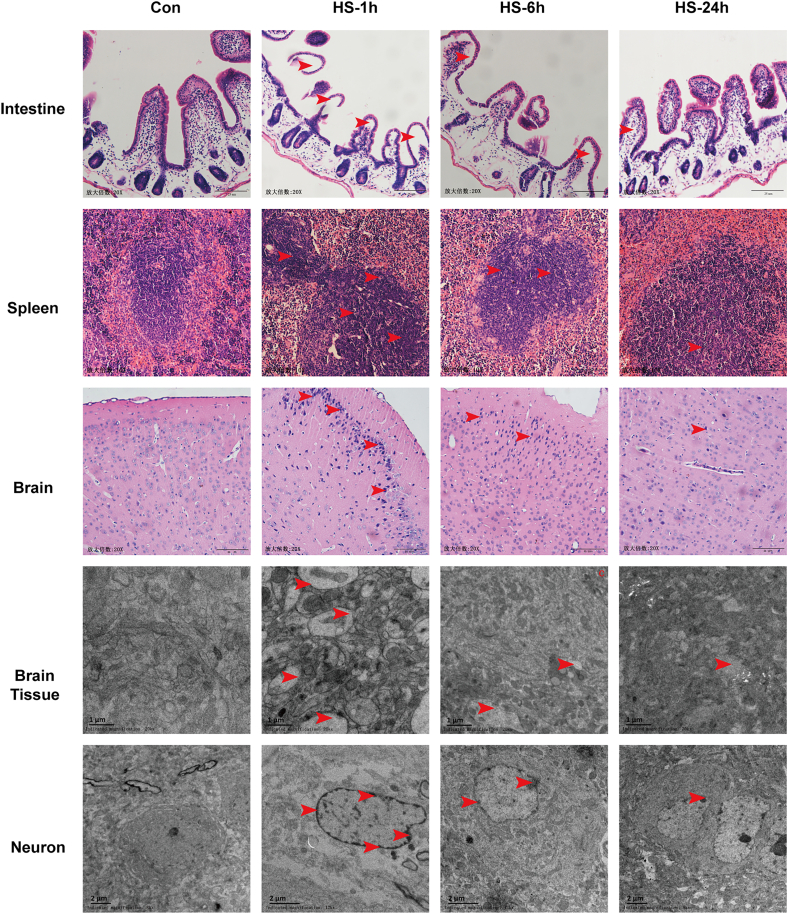
Time course of miltiorgan injury during the acute stage of heatstroke The mice were exposed to 41.2 ± 0.5°C ambient temperature until their rectal temperature reached 42.4°C, and then they were allowed to recover at an ambient temperature of 25 ± 0.5°C for the indicated times. Representative HE staining of the intestine, spleen, and brain, and TEM photomicrographs of brain sections at 1 h, 6 h, and 24 h post heat stress. The red arrow indicates the location of structural abnormalities.

We examined the histopathology of the intestine, spleen, and brain through HE staining. In the intestinal section, no significant abnormalities were observed in the control group; however, severe intestinal villous edema and a disordered structure of the lamina propria were detected in the HS-1 h group. Afterward, the intestinal lesions gradually improved, but little edema still can be found at 24 h after heatstroke (Figure 2). In the spleen sections, compared with those in the control group, partial cellular edema, nuclear hyperchromasia, an increased proportion of cytoplasmic vacuolization, and an irregular architecture of the splenic sinuses were detected in the HS-1 h group. At 24 h post-heatstroke, the spleen lesions still not recovered to normal levels, as evidenced by the irregular cell arrangement and morphology in the HS-24 h group (Figure 2). HE staining of brain sections from control mice revealed clear cytoplasm and nuclei in the cerebral cortex, while substantial cell swelling, nuclear pyknotic and vacuolation were observed in the HS-1 h group; even though these phenomena were ameliorated in the HS-6 h group, nuclear pyknotic was still obvious in some parts of the cerebral cortex. After 24 h of recovery, the brain tissue essentially returned to normal, and cells with condensed nuclei were present only occasionally in the HS-24 h group.

In addition to performing HE staining, we also evaluated the ultrastructure of the cerebral cortex by TEM. Compared to the control group, we found severe cell swelling accompanied by nuclear fragmentation and degeneration, in addition, destroyed endoplasmic reticulum and mitochondrial ridges could be seen in HS-1 h group. In HS-6 h group, although cell morphology was improved, edema and destroyed organelles could still be observed in neurons. Although the ultrastructure of the cerebral cortex was further improved in HS-24 h group but still cannot compare to the control group. All previous results suggest that multiple organ injury caused by heatstroke is most serious at 1 h after heatstroke onset, after which the tissue gradually recovers but it does not fully recover until 24 h post heatstroke.

### Time course of inflammation in peripheral circulation and the central nervous system during the acute stage of heatstroke

**Figure 3:**
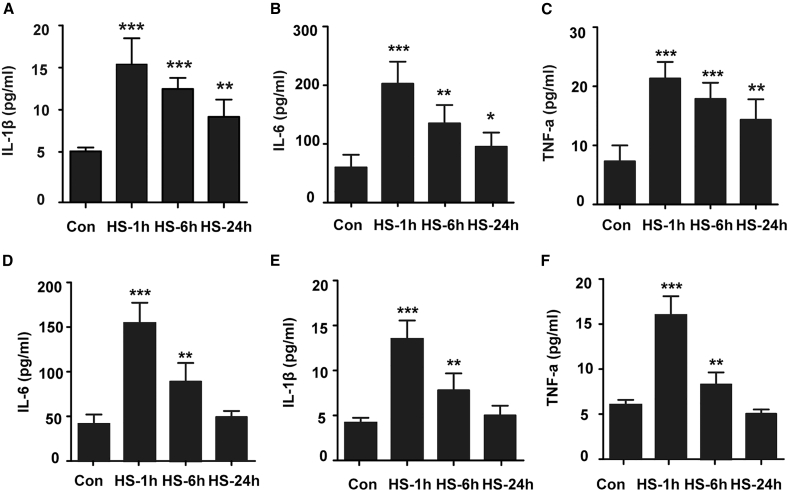
Time course of inflammation in peripheral circulation and the central nervous system during the acute stage of heatstroke The mice were exposed to 41.2 ± 0.5°C ambient temperature until their rectal temperature reached 42.4°C, and then they were allowed to recover at an ambient temperature of 25 ± 0.5°C for the indicated times. The protein levels of TNF-α, IL-1β, and IL-6 in serum (A–C) and cerebral cortex (D–F) of HS mice were assessed by ELISA. The data are expressed as the mean ± SEM of three independent experiments. ∗*p* < 0.05, ∗∗*p* < 0.01, ∗∗∗*p* < 0.001, vs. Con.

Inflammation is believed to cause multiorgan dysfunction; therefore, we investigated inflammation in both the peripheral circulation and the central nervous system during the acute state of heatstroke. We first examined the concentrations of TNF-α, IL-1β, and IL-6 in the serum of heatstroke mice and found that the trends of TNF-α, IL-1β, and IL-6 concentration were same; they all peaked at 1 h after heatstroke onset and then decreased gradually but were still significantly greater than those in the control group 24 h after heatstroke onset (Figures 3A–3C). We subsequently examined the expression of proinflammatory cytokines in cerebral cortex of heatstroke mice. The results revealed trends in TNF-α, IL-1β, and IL-6 concentrations within 6 h post heatstroke were consistent with the trends we detected in serum; all the concentrations peaked 1 h following heatstroke and then decreased gradually with increasing recovery time; however, a difference was apparent at 24 h post heatstroke, as demonstrated by the expression of TNF-α, IL-1β, and IL-6 we detected at cerebral cortex reduced to the level which was equal to that observed in the control group (Figures 3D–3F).

### Time course of functional damage to the intestinal barrier and blood-brain barrier during the acute stage of heatstroke

**Figure 4:**
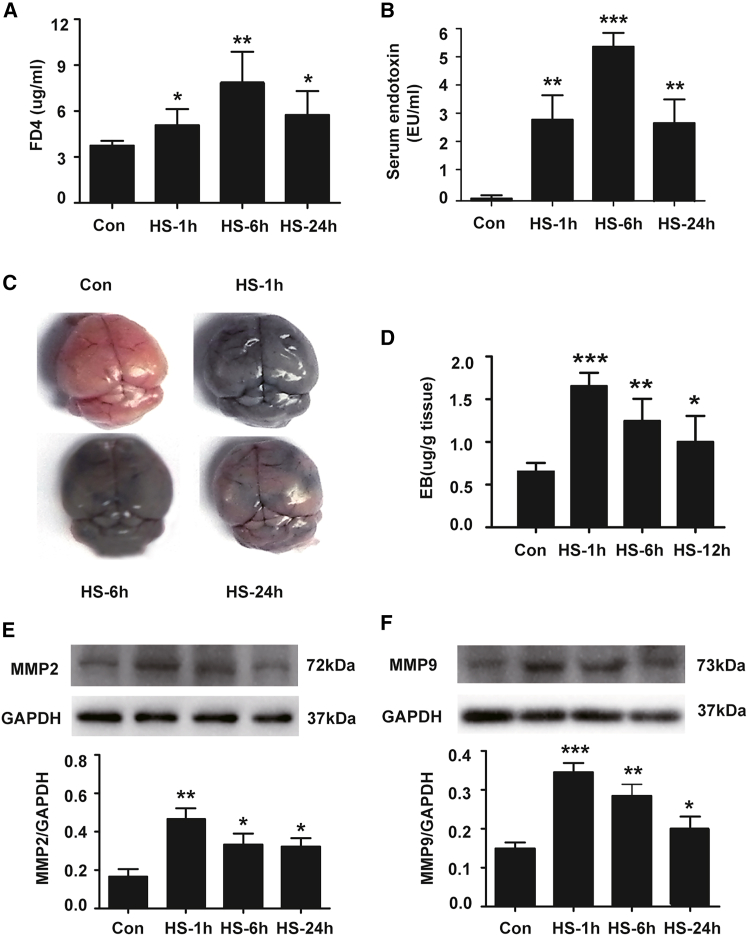
Time course of functional damage to the intestinal barrier and blood-brain barrier during the acute stage of heatstroke The mice were exposed to 41.2 ± 0.5°C ambient temperature until their rectal temperature reached 42.4°C, and then they were allowed to recover at an ambient temperature of 25 ± 0.5°C for the indicated times. (A) The concentration of FD-4 in serum was measured after its oral administration. (B) Serum endotoxin levels. (C) EB was injected into the mice via the tail vein after the onset of heatstroke, and representative images of EB leakage was shown. (D) Quantitative analysis of EB concentrations in brain tissues. (E) Expression of MMP-2 in the cerebral cortex at 1 h, 6 h, and 24 h post heatstroke. (F) Expression of MMP-9 in the cerebral cortex at 1 h, 6 h, and 24 h post heatstroke. The data are expressed as the mean ± SEM of three independent experiments. ∗*p* < 0.05, ∗∗*p* < 0.01, ∗∗∗*p* < 0.001, vs. Con.

The intestinal barrier and the BBB are critical structures for restraining the penetration of harmful inflammation-related substances such as endotoxins from the intestinal system into the systemic circulation and from the blood into the brain. Their increased permeability is related to systemic inflammation and neuroinflammation. We examined intestinal barrier integrity by assessing FD-4 and endotoxin concentrations in the serum of mice with or without heatstroke. We found that all heatstroke mice exhibited high intestinal permeability, as evidenced by the significantly higher concentrations of FD-4 and endotoxin detected in all heatstroke groups than in the control group (Figures 4A and 4B). We also evaluated cerebral microvasculature permeability by performing an evans blue extravasation assessment. The results revealed that the BBB was intact in the control group, while obvious extravasation of evans blue was observed in the brains of the HS-1 h group; afterward, the amount of EB in the cerebral cortex decreased but was still significantly greater than that in the control group (Figures 4C and 4D). The protein expression of MMP-2 and MMP-9 in the cerebral cortex also peaked 1 h after heatstroke onset but then gradually decreased but was still significantly higher than that in the control group until 24 h after heatstroke onset (Figures 4E and 4F). These results suggest that barriers in the intestine and brain are disrupted by heatstroke and that their permeability tends to decrease with increasing recovery time but still obvious 24 h post heatstroke in comparison to the control group.

### Time course of structural damage to both the intestinal barrier and blood-brain barrier during the acute stage of heatstroke

**Figure 5:**
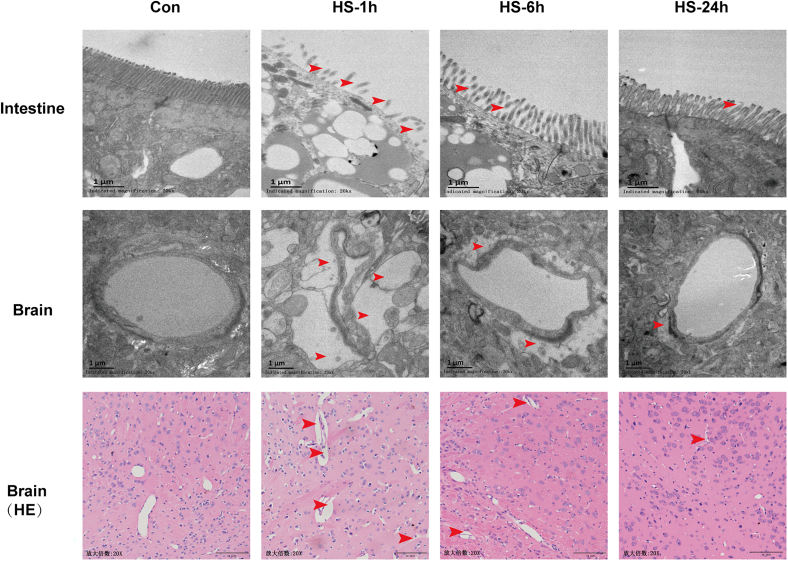
Time course of structural damage to both the intestinal barrier and blood-brain barrier during the acute stage of heatstroke The mice were exposed to 41.2 ± 0.5°C ambient temperature until their rectal temperature reached 42.4°C, and then they were allowed to recover at an ambient temperature of 25 ± 0.5°C for the indicated times. Representative TEM photomicrographs and HE staining of intestinal barrier and blood-brain barrier at 1 h, 6 h, and 24 h post heatstroke. The red arrow indicates the location of structural abnormalities.

We further assessed injury to both barriers by examining the ultrastructure of the intestine and brain with TEM. TEM of the intestine revealed many vacuoles in the intestinal wall accompanied by severe damage to the microvilli in the HS-1 h group. The damage to the intestine tended to be less severe in the HS-6 h group but was still damaged in comparison to the control group until 24 h after heatstroke onset (Figure 5). TEM images of cerebral blood vessels in the control group revealed that the vascular endothelial cells were intact, the basement membrane and podocytes were closely connected, and the lumen was smooth and without compression (Figure 5). In the HS-1 h group, the absence of endothelial cells and disconnection of basement membrane and foot cells were observed in some parts of the cerebral vessels, besides, severe extravascular edema compressed the wall of the vessels, even leading to occlusion of the lumen of the vessels could be seen in the HS-1 h group (Figure 5). Although the vascular morphology clearly improved in the HS-6 h group and the HS-24 h group, some edema was still detected in the HS-24 h group (Figure 5).

We also examined the vascular morphology in the cerebral cortex by HE staining. The results revealed good vessel morphology with intact vessel lumen and uniform smooth vessel walls in the control group, whereas severe compression/degeneration of the vascular lumen and extravascular edema were observed in the HS-1 h group; however, although compression/degeneration of the vascular lumen gradually decreased in the HS-6 h and HS-24 h groups, extravascular edema was still detected in the cerebral cortex of the mice in HS-24 h group (Figure 5). All the previous data indicated that with increasing recovery time, the structural damage to the barrier tends to decrease gradually.

### Time course of microglial phenotype during the acute stage of heatstroke

**Figure 6:**
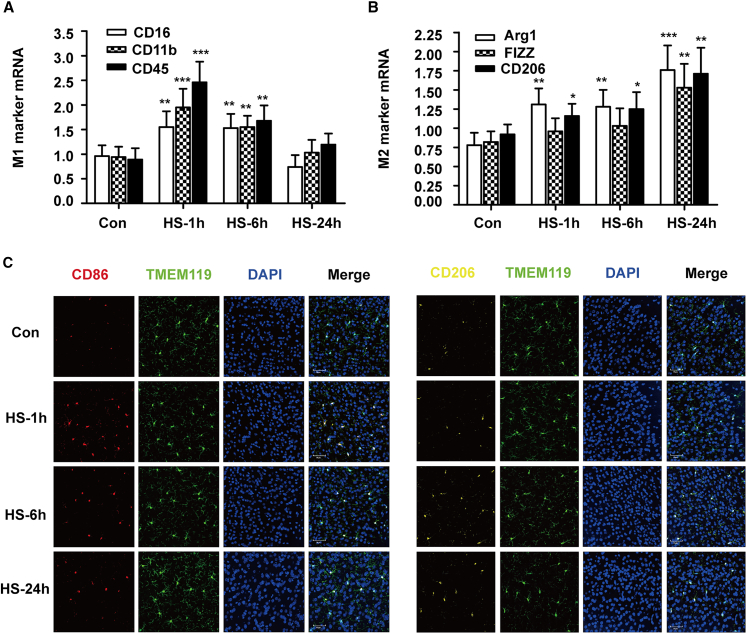
Phenotype of microglial activation in heatstroke mice The mice were exposed to 41.2 ± 0.5°C ambient temperature until their rectal temperature reached 42.4°C, and then they were allowed to recover at an ambient temperature of 25 ± 0.5°C for the indicated times. (A) The relative mRNA expression of classic microphage activation markers in the cerebral cortex was measured. (B) The relative mRNA expression of alternative microphage activation markers in the cerebral cortex was measured. (C) The phenotypes of microglia in the cerebral cortex were visualized by immunofluorescence staining for TMEM119 and CD86 or CD206. The data are expressed as the mean ± SEM of three independent experiments. ∗*p* < 0.05, ∗∗*p* < 0.01, ∗∗∗*p* < 0.001, vs. Con.

**Figure 7:**
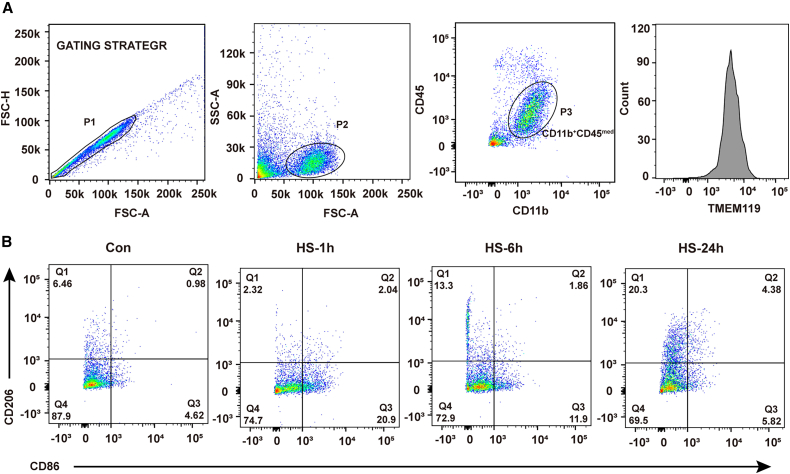
Flow cytometry analysis of microglial phenotype in mice The mice were exposed to 41.2 ± 0.5°C ambient temperature until their rectal temperature reached 42.4°C, and then they were allowed to recover at an ambient temperature of 25 ± 0.5°C for the indicated time. (A) Gating strategy for primary microglia from mouse brain. (B) The phenotypes of microglia in the cerebral cortex were analyzed by flow cytometry for CD86 and CD206 (5000 events in gate 3 were acquired).

Microglia play a critical role in clearing infiltrating pathogens, their phenotypic changes directly influence the outcomes of brain injury. To examined the phenotype of microglia in brain, we chose CD45, CD11b, and CD16 as classic activation (M1 phenotype) markers and CD206, FIZZ, and Arg1 as alternative activation (M2 phenotype) markers of microglia and then examined the expression of these different markers in the cerebral cortex of the mice by RT-qPCR. The results revealed that the trends in all the classic markers were the same: they peaked 1 h post heatstroke onset and then decreased gradually and almost returned to levels equal to those of the control group by 24 h post heatstroke onset (Figure 6A). However, the changes in the mRNA expression of alternative markers in microglia increased gradually and peaked 24 h after heatstroke onset, as evidenced by the high expression of CD206, FIZZ, and Arg1 in the HS-24 h group (Figure 6B). As resident macrophage in brain, microglia play a critical role in the neuroinflammatory response, and their phenotypic transformation has been reported to mediate nervous system damage and repair. To further characterized the phenotype of microglia in the cerebral cortex, confocal microscopy was used. Microglia were colocalized with TMEM119 and CD86 (M1 marker) or CD206 (M2 marker). The results showed that CD86 was highly localized in the HS-1 h group, whereas CD206 was highly localized in the HS-24 h group of heatstroke mice (Figure 6C). This result observed in immunofluorescence was further validated by flow cytometry (Figure 7). These results indicated that the phenotype of microglia in the cerebral cortex of heatstroke mice was mainly classic activation at 1 h post heatstroke onset; however, it had changed into alternative activation by 24 h post heatstroke onset.

### *In vitro* microglial activation phenotype

**Figure 8:**
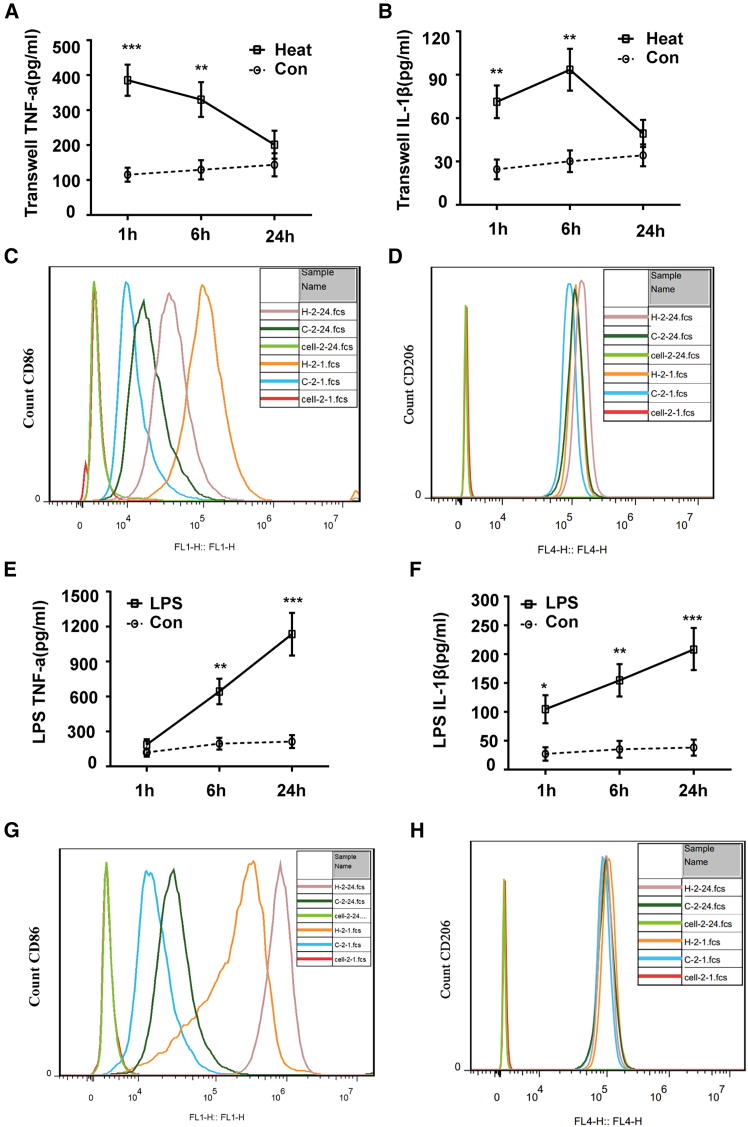
*In vitro* microglial activation phenotype BV2 cells were cocultured with N2a cells and then subjected to heat stress at 42°C for 2 h, followed by recovery at 37°C for the indicated times. (A and B) The secretion of TNF-α and IL-1β in the supernatant and (C–D) representative flow cytometry histograms for CD86 and CD206 expression, respectively. BV2 cells were pretreated with or without LPS, subjected to heat stress at 42°C for 2 h and allowed to recover at 37°C for the indicated times. (E and F) The secretion of TNF-α and IL-1β in the supernatant and (G and H) representative flow cytometry histograms for CD86 and CD206 expression, respectively. The data are expressed as the mean ± SEM of three independent experiments. ∗*p* < 0.05, ∗∗*p* < 0.01, ∗∗∗*p* < 0.001, vs. Con.

To further determine the phenotype of microglia during heat stress, cell experiments were performed. We cocultured BV2 cells (representing microglia) with N2a cells (representing neurons) in transwell plates to mimic the environment of microglia *in vivo* and then assessed the concentrations of TNF-α and IL-1β in the supernatant after the cells were subjected to heat stress treatment. Compared with those in the control group, the levels of both pro-inflammatory cytokines in the HS-1 h and HS-6 h groups were high; however, the concentrations of TNF-α and IL-1β in the HS-24 h group decreased almost to levels equal to those in the control group (Figures 8A and 8B). FACS analysis revealed that CD86 was highly expressed in the HS-1 h group and that CD206 was highly expressed in the HS-24 h group (Figures 8C and 8D). These results indicated that the phenotype of microglia after heat stress is classic activation 1 h post heat stress and alternative activation 24 h post heat stress. These results are consistent with our *in vivo* results.

LPS is the main component of endotoxin in the intestine and can leak through the damaged intestinal barrier and BBB and then activate microglia. We pretreated BV2 cells with LPS and then allowed them to respond to heat stress treatment, after which the TNF-α and IL-1β concentrations in the supernatant and the phenotype of the microglia were determined. The results revealed that the secretion of proinflammatory cytokines gradually increased beginning 1 h after LPS stimulation and peaked 24 h after LPS stimulation (Figures 8E and 8F). FACS analysis revealed that the phenotype of BV2 cells was classic activation, and no obvious alternative activation of microglia was observed in BV2 cells at any time point after LPS stimulation (Figures 8G and 8H). These results indicated that the phenotype of microglia 24 h after LPS and heat stress co-stimulation differed from the results we detected *in vivo*, which may imply that neuroinflammation 24 h post heatstroke is independent of peripheral inflammation.

## Discussion

Inflammation occurs in both the peripheral circulation and the brain during heatstroke and is usually correlated with multiorgan dysfunction and encephalopathy. In this study, we aimed to investigate how systemic inflammation and neuroinflammation develop during the acute stage of heatstroke and whether inflammation in the peripheral circulation can influence inflammation in the central nervous system during this period. We found that pathological injury in multiple organs, including the intestine, brain, and spleen, was severe 1 h after heatstroke and then gradually decreased with increasing recovery time; the expression of TNF-α, IL-6, and IL-1β in serum and intestinal barrier permeability peaked 1 h after heatstroke, after which they decreased gradually but remained at a significantly elevated level by 24 h compared with those in the control group. Changes in brain inflammation and BBB permeability 1 h and 6 h after heatstroke onset were similar to those of systemic inflammation and intestinal barrier breakdown; however, by 24 h they were different, with cessation of neuroinflammation but still high in systemic inflammation. Furthermore, we revealed that microglia exhibited classic activation 1 h and alternative activation 24 h post heatstroke onset. Taken together, our results suggest that systemic inflammation affects neuroinflammation within 6 h after heatstroke onset and neuroinflammation develops independently and is not influenced by circulation inflammation 24 h post heatstroke, possibly because of microglial phenotype transformation.

Over the past two decades, record-breaking heatwaves have increased the morbidity and mortality of heatstroke worldwide, resulting in the use of a large amount of medical resources and resulting in a great economic burden on society.^6^ Using mammals to establish heatstroke models can best imitate the pathophysiological development process of heatstroke in humans. Anesthetized mammalian heatstroke models have been used previously; however, some researchers have reported that in addition to the dissipation of heat by restricted animals through the cessation of autonomous behavior, anesthetic techniques can substantially lower the brain temperature by affecting cerebral blood flow and metabolism, two determinants of brain thermoregulation.^17^^,^^18^^,^^19^ Thus, conscious animals are more suitable for generating a heatstroke model; considering these findings, only awake and unrestrained mice have been widely accepted as animal models for experimental heatstroke in recent years.^19^ In the current study, we established a heatstroke model with conscious, unrestrained BALB/c mice, and monitored their rectal temperature during progression and recovery from heat stress. We found that the modeling time of heatstroke fluctuates from 200 to 250 min because of individual differences in mice; upon moving mice out of the heat chamber, their core body temperature gradually returns to normal after experiencing hypothermia. The thermoregulatory response profile during heat stress and recovery in this study was consistent with that reported by Leon,^20^^,^^21^ suggesting that we successfully established a heatstroke model in conscious, unrestrained mice.

Multiorgan system failure is the ultimate cause of death in heatstroke, which is a consequence of the combined effects of heat cytotoxicity, coagulopathies, and SIRS.^22^ A hot environment or strenuous exercise can elicit heat stress in the host; as a result of the thermoregulatory response, the cardiovascular system is activated.^23^^,^^24^ The primary cardiovascular response to heat stress is to increase blood flow in the skin to accelerate heat dissipation, whereas blood flow assigned to the splanchnic system and the brain decreases correspondingly to compensate for maintaining blood pressure within the acceptable or safe physiological range.^3^ Combined with heat cytotoxicity, persistent decreases in visceral and brain perfusion promote coagulation, SIRS and hypoxia in the brain, which together contribute to MODS in heatstroke.^7^ MODS may further deteriorate into multiple organ failure without appropriate and effective treatment. The brain, heart, liver, spleen, kidney, lungs, and intestine are the most vulnerable organs that are commonly affected during heatstroke, and different pathological injuries have been detected in these tissues; however, few studies have reported the progression of damage to these tissues after heatstroke. In this study, we examined histopathological changes in the intestine, spleen, and brain of mice at 1 h, 6 h, and 24 h after heatstroke onset and reported that the pathological injury was most severe at 1 h post heatstroke; afterward, the tissues gradually recovered but could still not be compared to that of mice in the control group until 24 h post heatstroke. Our results indicate that multiple organ injury occurred after heatstroke and did not fully resolve until 24 h after heatstroke onset.

SIRS was proposed by Bouchama as a critical origin of multiorgan system failure in heatstroke,^25^ and its formation mechanism has been clearly elucidated. In the preliminary stage of heatstroke, heat cytotoxicity and decreased visceral perfusion resulting from high-temperature exposure initiate damage to multiple organs. As the major organ of injury, the intestinal tract initiates SIRS.^26^ Severe pathological injury and a great deal of intestinal epithelium loss cause the intestinal barrier to become hyperpermeable; thereafter, the endotoxins in the intestinal flora enter the circulation and induce SIRS.^27^^,^^28^ In this study, dynamic changes in the systemic inflammatory response and intestinal damage in mice were observed within 24 h after heatstroke. Serum concentrations of TNF-α, IL-1β, and IL-6 and intestinal structural injury peaked by 1 h, after which they recovered gradually but remained significantly higher than those in control mice until 24 h post heatstroke. The changes in intestinal permeability were similar to the changes in serum cytokine levels and intestinal injury, as evidenced by obvious concentrations of FD-4 and endotoxins in mouse blood being detected until 24 h post heatstroke. Many researchers choose one time point during heatstroke to explore the mechanism of intestinal-mediated SIRS.^7^^,^^29^^,^^30^ In this study, we performed a preliminary examination of intestinal damage and the circulation inflammatory response for a longer time after heatstroke and found that as time progressed, the damage was gradually alleviated but was still evident in comparison to the control mice until 24 h after heatstroke onset.

As the core commander of the body, the brain is extraordinarily vulnerable to high temperature. Symptoms such as confusion, changes in consciousness, behavioral changes, and even more severe states, such as delirium and coma, are the diagnostic basis of heatstroke in the clinic.^5^^,^^31^ Neuroinflammation is thought to play a crucial role in aggravating brain injury and leading to neurological impairment in heatstroke. Accumulating evidence has indicated that BBB disruption is a common feature of neuroinflammation-mediated neurodegeneration.^32^ Increased BBB permeability can be detected when the brain temperature is higher than 38°C, after which intestinal and systemic toxins such as LPS can permeate into the brain parenchyma and induce neuroinflammation.^5^^,^^14^ In this study, we established a heatstroke model in mice whose terminal rectal temperature reached 42.4°C, and TNF-α, IL-1β, and IL-6 secretion and BBB integrity in the cerebral cortex were monitored at 1 h, 6 h, and 24 h post heatstroke. We found that cytokine expression nearly followed the same trend as that of BBB permeability; both were most severe 1 h after heatstroke onset and then recovered gradually with increasing recovery time. Increased neuroinflammation and a disrupted BBB after heatstroke in rodents have also been reported by other researchers^11^^,^^33^^,^^34^^,^^35^; unlike others, we observed changes in these processes during a longer recovery period after heatstroke onset in mice, and our results demonstrated that damage to the central nervous system caused by heatstroke tends to be alleviated with increasing recovery time.

Comprehensive analysis of some results in this study, including the changes in inflammation in the central nervous system and circulation as well as in BBB permeability, revealed that the changes were essentially consistent 1 h and 6 h after heatstroke onset; however, these phenomena changed at 24 h, as evidenced by the cessation of neuroinflammation but not of BBB permeability or the systemic inflammatory response. On the basis of these observations, we speculated that inflammation in the central nervous system may be affected by peripheral circulation due to increased BBB permeability within 6 h after heatstroke onset; however, neuroinflammation develops independently and is not influenced by inflammation in the circulation after 24 h of recovery.

Microglia are the resident macrophages in the brain, and their phenotype upon activation affects neuroinflammation and disease progression in the brain. We have recently reported that classic activation of microglia (also referred to as M1-like microglia) can trigger neuroinflammation and result in neurotoxicity and cognitive impairment, whereas alternative activation of microglia (also referred to as M2-like microglia) suppress inflammation and restore tissue function after heatstroke.^11^^,^^15^ BBB breakdown allows inflammation in the peripheral circulation to infiltrate the central nervous system and drive microglia toward reactive phenotypes. In a study using a mouse model that replicates pathogenic inflammation in systemic lupus erythematosus, microglia were shown to have both protective and toxic effects on maintaining BBB integrity upon persistent systemic inflammation attack.^36^ In this study, we also revealed that microglia play a dual role in the alteration of neuroinflammation in the presence of systemic inflammation in heatstroke, as demonstrated by classic activation 1 h post heatstroke and alternative activation 24 h post heatstroke; on the basis of these findings, we speculated that the change in the microglial phenotype from classic activation to alternative activation may explain why the neuroinflammation we detected in heatstroke mice peaked by 1 h and recovered by 24 h post heatstroke.

Besides microglia, the effect of blood-derived macrophages that migrate into the brain upon BBB breakdown on heatstroke-induced brain injury should also be considered. Similar to microglia, infiltrating macrophages also originate from the myeloid lineage and are capable of differentiating into pro and anti-inflammatory phenotypes in the brain. This shared characteristic makes them difficult to distinguish under pathological conditions. Tanaka et al. identified resident macrophages and blood-derived macrophages by using chimeric mice with enhanced green fluorescent protein bone marrow in ischemic brain injury. They found that GFP+ hematogenous macrophages were first detected on day 1, peaked in number by day 7 and subsequently declined, in contrast, GFP- resident microglial cells were rapidly activated by day 1 and microglial activation persisted until days 7.^37^ Other studies evidenced that monocytes-derived macrophages infiltrated into the brain during the early stage of stroke and differentiated into an anti-inflammatory phenotype in the mouse brain three days post ischemia^38^^,^^39^^,^^40^^,^^41^^.^ Combined the literatures with our results, we speculate that blood derived macrophages exacerbate the pro-inflammatory response of microglia during the early recovery stage (6 h) of heatstroke. However, by 24 h post heatstroke, the number of infiltrating macrophages declines as BBB disruption subsides, this reduction may facilitate a shift in microglial phenotype toward a predominant anti-inflammatory phenotype, thereby contributing to brain repair. Whether blood-derived macrophages are involved in brain injury and repair after heatstroke remains to be further investigated.

In conclusion, our results indicated that the severe brain injury that occurred in the early stage and during the heatstroke recovery period may have been a consequence of a combination of an inflammatory response in the peripheral circulation and the toxic effect of microglial activation. As the recovery time progressed, the microglial phenotype changed, which gradually resulted in neuroinflammation, and the brain then showed improvement during the acute stage of heatstroke. Microglial phenotype regulation may be a promising therapeutic strategy for heatstroke-related encephalopathy and needs further investigation.

### Limitations of the study

Two limitations should be noted in this study. One is that the molecular mechanism through which the microglial phenotype changes from classic activation to alternative activation during recovery after heatstroke have not been explored, and further research is needed. Another limitation is that only three time points during the 24 h post heatstroke were chosen to observe the relationship between neuroinflammation and systemic inflammation, and additional time points should be added to observe the changes in neuroinflammation and microglial phenotype transformation more accurately.

## Resource availability

### Lead contact

Requests for further information and resources should be directed to and will be fulfilled by the lead contact, Xuesen Yang (xuesenyyy@hotmail.com).

### Materials availability

This study did not generate new unique reagents.

### Data and code availability

• All data reported in this paper will be shared by the lead contact upon request.
• This paper does not report original code.
• Any additional information required to reanalyze the reported in this paper is available from the lead contact upon request.

## Acknowledgments

This work was supported by the 10.13039/501100005230Natural Science Foundation of Chongqing China (no. cstc2020jcyj-msxmX1020).

## Author contributions

Conceptualization: X.Y. and G.H.; methodology: P.L., Z.W., J.L., G.W., G.H., X.L., Z.L., and T.S; investigation: G.H., X.Y., P.L., and Z.W.; visualization: G.H., P.L., and Z.W.; supervision: G.H. and X.Y.; writing-original draft: P.L.; writing-review & editing: P.L., G.H., and X.Y.

## Declaration of interests

The authors declare no competing interests.

## STAR★Methods

### Key resources table

**Table:** 

REAGENT or RESOURCE	SOURCE	IDENTIFIER
**Antibodies**		

Mouse anti-GFAP	Lifetech	Cat# MA5-15738-1MG; RRID: AB_2537652
Rabbit anti-MMP2	ORIGENE	Cat# TA326260
Rabbit anti-MMP9	ORIGENE	Cat# TA326652
Rat anti-CD86	Abcam	Cat# ab119857; RRID: AB_10975549
Rabbit anti-CD206	Santa Cruze	Cat# sc-48758; RRID: AB_2266533
Mouse anti-Iba1	Abcam	Cat# ab283319; RRID: AB_2924797
Anti-rat Alexa Fluor 488	Thermo Invitrogen	Cat# A11006
Anti-rabbit Alexa Fluor 633	Sigma	Cat# SAB4600126
Rat anti-CD11b	Biolegend	Cat# 101245; RRID: AB_312788
Rat anti-CD45	Biolegend	Cat# 103113; RRID: AB_312978
Rat anti-TMEM119	eBioscience	Cat# 12-6119-82; RRID: AB_2848262
Rat anti-CD86	Biolegend	Cat# 159219; RRID: AB_3106043
Rat anti-CD206	Biolegend	Cat# 141707; RRID: AB_10896057
Rat anti-CD16/CD32	BD	Cat# 553141; RRID: AB_394656
Rabbit anti-TMEM119	Proteintech	Cat# 27585-1-AP; RRID: AB_2880915

**Chemicals, peptides, and recombinant proteins**		

TRIzol	Life Technologies	Cat#15596026
Formanide	Sigma	Cat#F9037-100ML
Evans blue dye	Sigma-Aldrich	Cat#E2129
PageRuler TM Prestained Protein Ladder	Thermo Scientific	Cat#26616
Clarity TM Western ECL Substrate	BIO-RAD	Cat#170-5060
FBS	GE Hyclone	Cat#SV30087.03
Penicillin-streptomycin	Beyotime	Cat#C0222
DMEM	Thermo gibco	Cat#C11995500BT
RIPA lysis buffer	Beyotime	Cat#P0013B
Goat serum	ZsBio	Cat#ZLI-9022
Liberase TL Research Grade	Sigma-Aldrich	5401020001
Deoxyribounclease 1 from bovine pancreas	Sigma-Aldrich	DN25
Percoll	Solarbio	P8370-100ml

**Critical commercial assays**		

QuantiCyto® Mouse IL-1β ELISA kit	eBioscience	Cat# 88-7013-22
QuantiCyto® Mouse TNF-α ELISA kit	eBioscience	Cat#88-7324-22
QuantiCyto® Mouse IL-6 ELISA kit	eBioscience	Cat#88-7064-22
Piercerm chromogenic Endotoxin Quan Kit	Thermo Fisher	Cat#A39552
PrimeScript TM RT reagent Kit with gDNA Eraser	TakaRa	Cat#RR047A
KAPA SYBR® FAST qPCR kit	Kapa Biosystems	Cat#KK4601
BCA Protein Quantification Kit	Yeasen	Cat#20201ES76

**Experimental models: Cell lines**		

BV2	Gifted by Neurology Department of Sourthwest Hospital (Chongqing, China)	N/A
N2a	Gifted by High altitude Medical Experimental Center (Chongqing, China)	N/A

**Software and algorithms**		

Image Lab	Bio-Rad	N/A
GraphPad Prism 5	GraphPad Software LLC	N/A
Zen 2	Zeiss	N/A
FlowJo V10	BD	N/A

### Experimental model and study participant details

#### Animals

Adult male BALB/c mice, 12 weeks of age, were provided by the Animal Centre of the Army Medical University (Chongqing, China). The animals were housed in a room maintained at 25±0.5°C and a humidity of 60±2% under a day/night cycle of 12 h for one week to acclimatize them to the laboratory environment. Both standard laboratory chow and water were provided ad libitum. This research was carried out in accordance with the recommendations in the Guide for the Care and Use of Laboratory Animals from the National Institutes of Health. All animal experiments were reviewed and approved by the Animal Ethical and Experimental Committee of the Army Medical University (Ethical approval number: AMUWEC2020113).

#### Cell culture and treatments

The immortalized murine microglial BV2 cells were a gift from the Neurology Department of Southwest Hospital (Chongqing, China). Cells were grown in DMEM (Gibco, USA) supplemented with 10% fetal bovine serum (FBS; HyClone, USA), 100 U/ml penicillin (Beyond, China) and 100 μg/ml streptomycin (Beyond, China). After the cells reached 80% confluence, they were seeded in six-well plates at 6 × 10^5^ cells/well and maintained overnight at 37°C in a humidified incubator containing 5% CO2. Then, the cells were subjected to heat stress at 42°C for 2 h, followed by recovery at 37°C for the indicated hours. In certain tests, cells were pretreated with 200 ng/ml LPS before they were subjected to heat stress.

### Method details

#### Experimental design

A computerized randomization procedure was used to assign BALB/c mice to two groups. The mice in the Con group were sham-heated at an ambient temperature of 25±0.5°C and a humidity of 60±2% with no food or water. The mice in the HS group were placed in an environment-controlled smart chamber (HOPE-MED 8150E, Tianjin, China) with an ambient temperature of 41.2±0.5°C and a humidity of 60±2% with no food or water until a core temperature of (Tc) >42.4°C was achieved, indicating the onset of heatstroke [17]. They were then transferred to an environment where the ambient temperature and humidity were the same as those of the Con group, and they were given free access to food and water to recover for the indicated hours. Tc was monitored with a digital thermometer (ALC-ET06; Shanghai Alcott Biotech Co., Shanghai, China), which was inserted 2 cm into the rectum at the indicated times. The body weights of the mice were determined before heat exposure and sacrifice. The mice were observed for 24 hours to calculate their survival rate after heatstroke.

#### Hematoxylin and eosin (HE) staining

Samples of the whole brain and the ileal segment 5 cm away from the ileocecal valve were separated and fixed in 4% paraformaldehyde after the mice were anesthetized with an intraperitoneal injection of sodium pentobarbital (40 mg/kg). The fixed tissues were embedded, sectioned, stained and observed under an optical microscope (Leica, Germany).

#### BBB permeability assay

BBB permeability was assessed by detecting cerebral Evans blue leakage. Evans blue dye (4% in saline, 4 ml/kg; Sigma–Aldrich) was injected into the mice via the tail vein after the onset of heatstroke. Then, the mice were anesthetized with sodium pentobarbital (40 mg/kg) and their whole brains were removed. After being homogenized in formamide (1 ml/100 mg, beyond), the brain samples were incubated at 60°C for 24 h. The Evans blue concentration in the supernatant was measured at 620 nm with a spectrometer (Bio-Rad, TECAN) and finally quantified according to the standard curve.

#### Real-Time PCR

Total RNA was extracted with TRIzol® reagent (Invitrogen, Carlsbad, CA, USA), and the concentration and quality of the RNA were measured. In accordance with the manufacturer’s instructions, total RNA was reverse transcribed into cDNA using the PrimeScriptTM RT reagent Kit with gDNA Eraser and then amplified and detected by using a KAPA SYBR® FAST qPCR kit (Kapa Biosystems, Boston, MA, USA) and the Bio-Rad CFX 96TM Real-Time PCR Detection System (Bio-Rad). The primers used were as follows: CD16: Fwd 5'- TTG CTT TTG CAG ACA GGC AG -3'; Rev 5'- TTC GCA CAT CAG TGT CAC CA-3'. CD11b: Fwd 5'- AAG GAT TCA GCA AGC CAG AA-3'; Rev 5'- TAG CAG GAA AGA TGG GAT GG-3'. CD45: Fwd 5'- ATC ATC GCC AGC ATC TAT CC-3'; Rev 5'- GAC GGA CAC AGT TAG CAT CC-3'. Arg1: Fwd 5'- GAC CTG GCC TTT GTT GAT GT-3'; Rev 5'- CCA TTC TTC TGG ACC TCT GC-3'. FIZZ: Fwd 5'- TGC CAA TCC AGC TAA CTA TCC-3'; Rev 5'- CAG TAG CAG TCA TCC CAG CA-3'. CD206: Fwd 5'- GGG ACT CTG GAT TGG ACT CA-3'; Rev 5'- CCA GGC TCT GAT GAT GGA CT-3'. HPRT: Fwd 5'-GTT AAG CAG TAC AGC CCC AAA-3'; Rev 5'-AGG GCA TAT CCA ACA ACA AAC TT-3'.

#### Enzyme-Linked immunosorbent assay

Cerebral cortex tissue was obtained after the mice were deeply anesthetized with sodium pentobarbital (40 mg/kg) and then homogenized in precooled PBS. The supernatant or medium of the homogenate or cells was collected after centrifugation. The concentrations of TNF-α, IL-1β and IL-6 in the tissue lysates were determined using commercial ELISA kits (eBioscience, Thermo Fisher Scientific, Inc.) in accordance with the respective protocols.

#### Transmission electron microscopy

Brain tissue perfusion fixation was performed at room temperature after the mice were deeply anesthetized with sodium pentobarbital (40 mg/kg). A 1 mm3 cortical tissue block was harvested from the brain and then fixed by immersion in 2.5% glutaraldehyde at 4°C overnight. The samples were subsequently postfixed with 1% osmium tetroxide and 1.5% potassium ferrocyanide, dehydrated and embedded in epoxy resin. Afterward, the samples were cut into 80-nm-thick ultrathin sections and stained with 4% uranyl acetate lead citrate. The tissue ultrastructure was viewed with a transmission electron microscope (TECNA110; FEI, USA).

#### Western blotting

Cerebral cortex tissue was obtained after the mice were deeply anesthetized with sodium pentobarbital (40 mg/kg) and then lysed in precooled RIPA lysis buffer (Beyotime Company, Jiangsu, China) supplemented with a cocktail of phosphatase and protease inhibitors (Roche Diagnostics). Total protein was collected by centrifugation at 13000 × g for 15 minutes at 4°C. Thirty micrograms of extracted total cellular protein was separated by 10% SDS‒polyacrylamide gel electrophoresis (SDS‒PAGE) and electrophoretically transferred to a 0.2 μm nitrocellulose membrane (Bio-Rad, CA, USA). The membranes were then blocked with 5% nonfat milk for 1 h at 25°C. The membrane was further incubated overnight at 4°C with primary antibodies against MMP9 (TA326652; ORIGENE, USA), MMP2 (TA326260; ORIGENE, USA) and glyceraldehyde-3-phosphate dehydrogenase (GAPDH; 2118L; CST, USA). This was followed by incubation with the corresponding secondary antibody (ZB2301, ZB2305, ZsBio, China) for 2 h at 25°C. The protein signal was detected by using enhanced chemiluminescence reagent (Bio-Rad, USA), and the intensity was captured with a ChemiDoc MP gel imaging system (Bio-Rad, USA).

#### Immunofluorescence staining

Brain tissue perfusion fixation was performed at room temperature after the mice were deeply anesthetized with sodium pentobarbital (40 mg/kg). After being postfixed with 4% paraformaldehyde, sections were cut with a frozen slicer (CM190; Leica, Germany) and blocked with goat serum (ZsBio, China), after which they were incubated with anti-rabbit TMEM119(27585-1-AP; Proteintech, China), mouse anti-rat CD86 (ab119857; abcam, USA), and goat anti-mouse CD206 (sc-58986; Santa Cruz, USA) antibodies at 4°C overnight. Following incubation with the corresponding secondary antibodies, namely, chicken anti-goat IgG (H + L) CF633 (SAB4600124; Sigma‒Aldrich, USA), rabbit anti-mouse IgG (H + L) Alexa Fluor 488 (A11059; Lifetech, USA), and monkey anti-rabbit IgG (H + L) CF568 (SAB4600076; Sigma‒Aldrich, USA), the sections were photographed under an LSM 880 confocal laser scanning microscope (Carl Zeiss GmbH, Jena, Germany) after staining the nuclei with 4′,6-diamidino-2-phenylindole (DAPI; Beyotime Biotechnology, China).

#### Primary microglia isolation and flow cytometry

Microglia isolation and flow cytometry analyzation were mainly referenced to a previously published protocol.^42^ Briefly, mice were deeply anesthetized with sodium pentobarbital (40 mg/kg) at certain time points post heatstroke, after transcardially perfused with cold PBS, the cerebral cortex were harvested and placed in cold digestion cocktail which contain Liberase (1,6 wunsch/ml, Sigma Aldrich, St. Louis, Missouri, MO, USA) and Deoxyribonuclease 1 (0.5mg/ml, Sigma Aldrich, St. Louis, Missouri, MO, USA) in PBS. Cut the sample into pieces with tissue scissors and incubated for 30 min at 37°C, after digestion, the single-cell suspensions were obtained by passing the brain homogenate through a 70-um cell filters. After removing debris by centrifuging, the cell pellets were resuspended in 30% isotonic Percoll solution, slowly underlaid 37% isotonic Percoll solution and 70% isotonic Percoll solution to the bottom in sequence, centrifuge the gradient with 800g at room temperature for 40 min with minimal or no brake. Collected microglia-enriched cell population at the border between the 37% and 70% Percoll gradient and washed with cold PBS to remove the residual Percoll. The obtained cells were blocked with mouse Fc Block (1:100, CD16/CD32, BD, Franklin Lakes, NJ, USA) prior to incubating with a mixture of antibodies against CD11b-FITC (1:100; Biolegend, San Diego, CA, USA), CD45-PE-Cy7(1:100; Biolegend, San Diego, CA, USA), TMEM119-PE (1:50, eBioscience, San Diego, CA, USA), CD86-FITC (1:100; Biolegend, San Diego, CA, USA) and CD206- APC (1:100; Biolegend, San Diego, CA, USA) at 4°C for 30 min. After incubation, the samples were washed and transferred into FACS tubes, data were collected on a BD Canto flow cytometer (BD Biosciences, San Jose, CA, USA).

#### Flow cytometric analysis of BV2 cells

The microglial polarization markers CD206 and CD86 were detected by flow cytometry. The cells were collected and washed three times with PBS, blocked with goat serum and incubated with the rat anti-mouse antibody CD206-APC (1:100; Biolegend, San Diego, CA, USA) or the rat anti-mouse antibody CD86-FITC (1:100; Biolegend, San Diego, CA, USA) for 1 h at 4°C in the dark. Then, the cells were resuspended in PBS containing 0.1% azide nanoparticles and 1% bovine serum albumin, adjusted to a density of 1x10^6^/mL and detected on a BD Accuri C6 flow cytometer (BD Biosciences, San Jose, CA, USA).

#### Serum endotoxin analysis

A Piercerm Chromogenic Endotoxin Quan Kit (Thermo Fisher Scientific, USA) was used for the endotoxin bioassay in accordance with the manufacturer’s instructions. All the materials used in this experiment were nonpyrogenic. The plasma samples and amebocyte lysate reagent were gently mixed for 20 min at 37°C, chromogenic substrate solution was added, and the mixture was incubated at 37°C for 6 min. Twenty-five percent acetic acid was added to the tube to stop the reaction, and the signals were detected at 405 nm in a microplate spectrophotometer.

#### FITC-dextran 4,000 Da (FD-4) detection

FD-4 (25 mg/mL; Sigma Chemical Co.) working fluid was prepared in the dark according to the manufacturer’s instructions, and gastric lavage was performed according to the weight of the mice (400 μL/30 g). The fluorescence signal of FD-4 in plasma was captured by fluorescence spectroscopy (Varioskan LUk, Thermo Scientific) with an excitation wavelength of 485 nm and an emission wavelength of 535 nm. The plasma extracted from the control group was used as a negative control, and a serial dilution of FITC was used to construct a standard curve.

### Quantification and statistical analysis

The data are presented as the means ± standard errors. GraphPad Prism was used to perform one-way ANOVA for multiple variables, and an unpaired Student’s t test was used to compare two groups. P<0.05 or less was considered to indicate statistical significance.
