# Supplementary material for: Changes in the microglial phenotype drive neuroinflammation independent of systemic inflammation in the acute stage of heatstroke
Source: iScience. 2026 Mar 6;29(4):115254. doi: 10.1016/j.isci.2026.115254 (PMC13058986; doi:10.1016/j.isci.2026.115254)

iScience, Volume *29*

Supplemental information

Changes in the microglial phenotype drive neuroinflammation independent of systemic inflammation in the acute stage of heatstroke

Ping Li, Zeze Wang, Jun Liu, Gong Wang, Xue Luo, Zhen Luo, Tingting Shen, Genlin He, and Xuesen Yang

Figure S1. Original image of the Western blot data in Figure 5

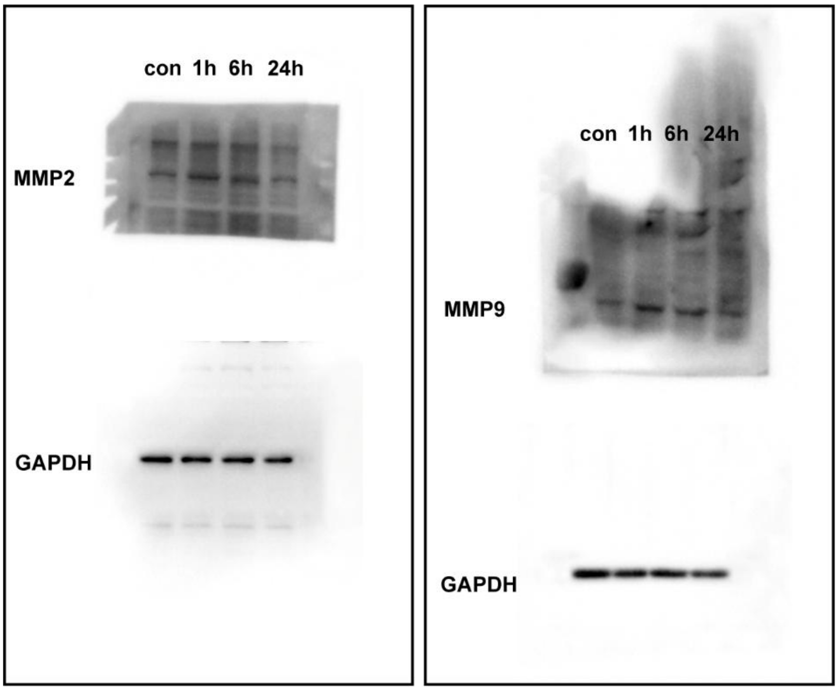

Supplement: Document S1. Figure S1 [file mmc1.pdf]
